# Impact of Anterior Margin Status Following Skin- or Nipple-Sparing Mastectomy in Patients Undergoing Implant-Based Breast Reconstruction

**DOI:** 10.1245/s10434-025-18982-3

**Published:** 2026-01-31

**Authors:** Nicole Dorothea Taylor, Angela Rezo, Usama Majeed, Yi Mike He, Rebecca Louise Read

**Affiliations:** 1 Department of Surgery, North Canberra Hospital, Bruce, ACT Australia; 2 Australian National University, Acton, ACT Australia; 3 Radiation Oncology Department, The Canberra Hospital, Garran, ACT Australia

**Keywords:** Anterior margin, Superficial margin, Skin-sparing, Nipple-sparing, Mastectomy, Implant, Radiotherapy, Recurrence

## Abstract

**Background:**

This study aims to increase the evidence guiding the management of positive or close anterior margins for invasive breast cancer or ductal carcinoma in situ after skin- or nipple-sparing mastectomy (SSM/NSM) and implant-based breast reconstruction. Re-excision to clear margins has been recommended but is not always feasible. Post-mastectomy radiotherapy may reduce rates of recurrence but also adversely impacts reconstruction outcomes.

**Methods:**

A total of 160 patients undergoing SSM/NSM with immediate direct-to-implant- or two-stage expander-to-implant-based breast reconstruction between March 2012 and September 2022 were retrospectively identified. Final margin status and adjuvant therapies were recorded. Primary outcomes assessed were ipsilateral, regional, and distant recurrence rates. Implant loss rates were assessed as a secondary outcome. Analysis of recurrence rates were performed based on margin status and adjusting for the confounding effects of radiotherapy, chemotherapy, and endocrine treatments.

**Results:**

Positive or close margins were identified in 19% (*n*=30) and 11% (*n*=18) of patients, respectively. The median follow-up period was 60 months. The ipsilateral breast cancer recurrence rate was 6% (*n*=9), the regional recurrence rate was 3% (*n*=5), and distant metastasis occurred in 6% (*n*=10). No statistically significant association was found between margin status and recurrence rates. When the anterior margin was positive or close for cancer or ductal carcinoma in situ, the addition of radiotherapy reduced the rates of ipsilateral breast cancer recurrence (odds ratio 0.08; 95% confidence interval 0.01–0.71; *p*=0.02). Radiotherapy was associated with increased overall rates of unplanned exchange or loss of the initial implant over the study period (relative risk 1.65; 95% confidence interval 1.23–2.22, *p*<0.001).

**Discussion:**

Radiotherapy may provide a safe oncologic alternative to management of close anterior margins after SSM/NSM when re-excision is not possible, but it results in increased implant-related complications.

Skin- or nipple-sparing mastectomy (SSM/NSM) has been regarded as oncologically safe in appropriately selected patients and facilitates immediate breast reconstruction.^1^ This can be beneficial for patients as it may provide a preferred aesthetic outcome and contribute to their overall wellbeing.^2^ However, oncological safety should not be compromised.^3^

Pathologically, invasive breast cancer margins are classified as “involved” if there is cancer at the edge of a specimen or “close” if there is cancer within a defined distance from the edge of the specimen.^4^ Adequate margins in breast cancer surgery have been a topic of controversy,^5^ and recommendations vary around the world. The data around adequate margins for mastectomy are generally inferred from the breast conservation surgery literature. The American Society for Radiation Oncology,^6^ Society for Surgical Oncology, European Society for Medical Oncology,^7,8^ and United States National Comprehensive Care Network (US NCCN) agree that margins of 2 mm are required for ductal carcinoma in situ (DCIS) given its histologic propensity for spread via skip lesions along ducts. Recommendations for invasive carcinoma generally state that there should be no tumor on ink. However, a recent meta-analysis suggested this was inadequate.^4^ The 2024 UK Association of Breast Surgery guidelines^9^ and the recent UK National Institute for Health and Care Excellence guidelines recommend margins of at least 1 mm for invasive carcinoma.^10^

When a mastectomy is performed, the superficial and deep margins correspond to the anatomical boundaries of the breast glandular tissue, which meet circumferentially. The superficial margin follows the pre-mammary plane, encompassing all areas where the breast glandular tissue interfaces with the lipodermal layer.^11^ The deep margin is mostly on the pectoral fascia. It is generally assumed that dissection along these planes removes all breast tissue. Consequently, these margins are not routinely re-excised,^12^ and the oncologic significance of positive or close superficial (anterior) margins remains a subject of ongoing debate.

A 2020 systematic review and meta-analysis involving patients managed with all types of mastectomy concluded that positive margins may increase the risk of local recurrence (LR) and distant recurrence. Patients with involved or close margins and who were not managed with radiotherapy had an increased risk of LR (relative risk [RR] 2.6).^13^ Another retrospective study of mastectomy outcomes without adjuvant radiotherapy in 440 patients managed with SSM/NSM showed that the overall 5-year risk of LR was higher in patients with an initial margin <2 mm than for those with margins >2 mm (11.2 vs 3.1%).^5^

Post-mastectomy radiotherapy (PMRT) is not routinely recommended but is indicated for patients at increased risk of recurrence. Current NCCN guidelines incorporate margin status into recommendations for PMRT after SSM/NSM.^14^ For patients with involved margins, re-excision to negative margins is preferred; however, if this is not feasible, PMRT to the chest wall and regional nodes should be strongly considered.^14^

PMRT is known to increase the rate of complications associated with implant-based breast reconstruction, including implant loss, capsular contracture, and reduced patient satisfaction.^15^ We were particularly interested in the management of involved or close margins in this setting because, although PMRT may mitigate potential poor oncological sequelae, it also has a significant impact on the results of implant-based reconstruction.

This study aims to increase the evidence base guiding management when final histology following SSM/NSM shows involved or close anterior margins, particularly regarding the use of adjuvant radiotherapy.

## Methods

This observational study included a retrospective review of all patients undergoing SSM/NSM in combination with implant-based breast reconstruction at North Canberra Hospital (formerly Calvary Public Hospital, Bruce), in the Australian Capital Territory (ACT), Australia. The study was approved by the ACT Health Research Ethics and Governance Office, which waived the requirement for informed consent because of the low-risk, retrospective design. All patients undergoing surgery between March 2012 and September 2022 were identified following review of all procedures performed. There were no age restrictions. Patients were included in the study if they underwent SSM/NSM with implant-based breast reconstruction for either invasive breast cancer or DCIS. Patients were offered appropriate neoadjuvant and adjuvant therapies as recommended by the ACT Health Breast Cancer Multidisciplinary team. Patients were excluded if they underwent prophylactic risk-reducing breast surgery where no cancer or DCIS was identified, if their initial surgery was in another jurisdiction, if they had previously been diagnosed with breast cancer, or if they had inflammatory breast cancer.

During this study period, our unit included four breast surgeons who hold fellowships in General Surgery with the Royal Australasian College of Surgeons. All completed post-fellowship training in oncoplastic breast surgery and are accredited with the Society of Breast Surgeons of Australia and New Zealand. All perform implant-based breast reconstruction. Tissue-based breast reconstruction is performed by the Plastic and Reconstructive Surgery Department at another hospital. Access to immediate tissue-based reconstruction is limited, and this surgery is not routinely performed when PMRT is likely to be required. Consequently, tissue-based breast reconstruction is usually undertaken as a delayed procedure.

Specimen orientation and pathology reports were standardized to include routine reporting of anterior, posterior, and four radial margins. For the purposes of this study, “anterior” margins were defined as the closest histologic anterior or radial margin. Margins were considered involved if there was invasive carcinoma or DCIS at the margin (0 mm, “tumor-on-ink”). Margins were considered close if they were <1 mm for invasive carcinoma or <2 mm for DCIS. Margins recorded were final surgical margins for any patients undergoing re-excision. The site of the involved or close margin(s) were identified by correlating the oriented surgical specimen and pathology. If re-excision was performed, it was completed over a broad front. If surgery was performed for a positive or close margin below the nipple areola complex, the entire complex and residual deep tissue was excised.

The primary outcome was ipsilateral breast cancer recurrence. Secondary outcomes included regional recurrence, distant metastasis, and implant loss.

Statistical analysis was performed using Jamovi version 2.6.44.^16^ Exploratory binary analysis using the Chi-squared test for comparing two independent proportions was performed to assess relationships between margin status and outcome. To detect potential confounders, binomial logistic regression analysis was utilized; 95% confidence intervals (CIs) are reported.

## Results

In total, 160 patients managed with SSM/NSM during the study period were identified. The median age was 48 years (range 25–82). All were female. Two had previous implants for augmentation purposes.

Of the 160 patients, 12 (8%) were lost to follow-up. Mean follow-up was 64 months (standard deviation 34 months; median 60 months [range 3–160], interquartile range 33 months). Mastectomy was performed via a skin-sparing approach in 42.5% (*n*=68) and a nipple-sparing approach in 57.5% (*n*=92). Ethnicity was categorized according to the Australian standard. Clinicopathologic details are summarized in Table 1.^17^

**Table 1:** Clinicopathologic details of patients undergoing skin- or nipple-sparing mastectomy and immediate implant-based reconstruction for breast cancer

Characteristic	Total *n*=160 (100%)	Surgery first *n*=129 (100%)	Neoadjuvant chemotherapy *n*=31 (100%)
Ethnicity			
Australian	104 (65)		
Aboriginal and Torres straight Islanders	1 (1)		
Oceania	3 (2)		
European	15 (9)		
Asia	16 (10)		
Americas	5 (3)		
Africa and Middle East	4 (3)		
Not stated	12 (8)		
Histology			
DCIS only	24 (15)	24 (19)	0 (0)
IDC	107 (67)	77 (60)	30 (97)
ILC	22 (14)	21 (16)	1 (3)
IDC/ILC mixed	3 (2)	3 (2)	0 (0)
Other invasive	4 (3)	4 (3)	0 (0)
Multifocal^b^	72 (45)	59 (46)	13 (42)
LVI^b^	48 (30)	44 (34)	4 (13)
T Category		pT	ypT
Tis (ypT0/is)		24 (19)	9 (29)
T1 (ypT1)		40 (31)	14 (45)
T2 (ypT2)		51 (40)	6 (19)
T3 (ypT3)		14 (11)	2 (6)
T4 (ypT4)		0 (0)	0 (0)
Nodal status		pN	ypN
N0		69 (53)	20 (65)
N1		48 (37)	8 (26)
N2		7 (5)	3 (10)
N3		5 (4)	0 ()
Grade (invasive carcinoma)^b,c^			
1		13 (10)	2 (6)
2		52 (40)	13 (42)
3		39 (30)	16 (52)
Biologic subtype^b,c^			
ER+/PR+/Her2-(Ki 67 <15%)	28 (18)	28 (22)	0 (0)
ER+/PR+/Her2-(Ki 67 ≥15%)	67 (42)	54 (42)	13 (42)
HER2+	26 (16)	17 (13)	9 (29)
TNBC	13 (8)	4 (3)	9 (29)

Data are presented as *n* (%); North Canberra Hospital, Australian Capital Territory, Australia; March 2012 to September 2022

DCIS, ductal carcinoma in situ; ER, estrogen receptor; HER2, human epidermal growth factor receptor 2; IDC, invasive ductal carcinoma; ILC, invasive lobular carcinoma; LVI, lymphovascular invasion; PR, progesterone receptor; TNBC, triple-negative breast cancer ^a^Values missing for two patients with recurrence where axillary status not assessed

^b^Multifocal disease, LVI, grade, and molecular subtype based on pre-treatment biopsy and imaging

^c^Values missing where information not available (i.e. grade, Ki 67)

### Margins

Final histology returned negative margins in 112 patients (70%). Positive anterior margins for invasive breast cancer and/or DCIS were found in 30 patients (19%). Overall, 48 patients (30%) had close and/or positive margins in this series. Results are summarized in Table 2.

**Table 2:** Margin status

Margin^a^	Pathology	Patients *n* = 160 (100)
Negative (*n* = 112)	Both invasive and DCIS	112 (70)
Positive (*n* = 26)	Invasive	10 (6)
	DCIS	10 (6)
	Both invasive and DCIS	6 (4)
Close (*n* = 18)	Invasive	8 (5)
	DCIS	9 (6)
	Both invasive and DCIS	1 (1)
Positive and close (*n* = 4)	Invasive positive and DCIS close	2 (1)
	Invasive close and DCIS positive	2 (1)

Final histologic margins in patients undergoing skin- or nipple-sparing mastectomy and immediate implant-based reconstruction for breast cancer at North Canberra Hospital, Australian Capital Territory, Australia; March 2012 to September 2022. Data are presented as *n* (%); DCIS, ductal carcinoma in situ

^a^For invasive carcinoma and DCIS, positive margins were 0 mm. For invasive carcinoma, close margins were <1 mm. For DCIS, close margins were <2 mm

One patient had pleomorphic LCIS at the margin, and this was recorded in the DCIS group given the similarities in management.

Three patients had positive margins at the time of the initial SSM/NSM and returned to the operating theatre. Magnetic resonance imaging (MRI) was not used to guide margin re-excision in any of these patients. The re-excision specimens included the nipple and superficial flap in one patient. The specimen weights were 90 g, 36 g, and 120 g for the three patients, respectively. No further invasive or in situ malignancy was seen in any of these re-excision specimens, and the margins were considered negative after re-excision.

### Adjuvant Treatment

Adjuvant radiotherapy was administered in 51% of patients (*n*=81). Chemotherapy was administered in 67% of patients (*n*=107). Of the 107 patients receiving chemotherapy, it was administered in the neoadjuvant setting in 31 patients (29%) and between an initial failed breast conservation surgery and completion SSM/NSM in eight patients (7%). The pathologic complete response rate was 19% (*n*=6).

### Outcomes

During the study, six patients died from all causes, with breast cancer-specific mortality in three patients.

Recurrences, including ipsilateral recurrence, regional nodal recurrence, and distant metastatic disease, were identified in 20 patients (13%). Nine of these patients had ipsilateral recurrence, one of whom also had intercurrent distant metastasis at the time of identification of recurrence. Five patients had regional nodal recurrence, and all except one of those patients also had distant metastasis identified within the year of identification of recurrence. Five patients developed distant recurrence in the absence of locoregional recurrence. One patient developed a contralateral breast cancer years after initial SSM for DCIS.

### Margin Status and Recurrence

In six of the 20 (30%) patients who experienced any recurrence event, margins were involved or close for cancer or DCIS. In the other 70% (14/20) of patients who experienced any type of recurrence, margins were negative. The overall rates of locoregional recurrence were 10% (5/48) in those with positive or close margins and 8% (9/112) in those with negative margins. Chi-squared tests for comparing two independent proportions were performed to assess the association between margin status and recurrence outcomes. No statistically significant association was observed, as summarized in Table 3.

**Table 3:** Margin and subsequent breast cancer recurrence

Margin and recurrence	Ipsilateral recurrence	Regional recurrence	Distant metastasis	Any recurrence
Invasive margin positive	0.99 (0.89–1.13; 0.98)	0.97 (0.93–1.00; 0.42)	0.99 (0.88–1.12; 0.89)	0.98 (0.82–1.17; 0.84)
Invasive margin close	1.04 (0.86–1.26; 0.61)	1.07 (0.90– 1.29; 0.24)	1.16 (0.87–1.53; 0.09)	1.07 (0.81–1.43; 0.56)
Invasive margin close or positive	1.02 (0.91–1.13; 0.75)	1.00 (0.93–1.08; 0.92)	1. 06 (0.93–1.20; 0.32)	1.02 (0.87–1.19; 0.83)
DCIS margin positive	0.99 (0.89–1.13; 0.98)	1.03 (0.92–1.15; 0.53)	1.06 (0.90–1.26; 0.37)	1.06 (0.85–1.31; 0.58)
DCIS margin close	1.03 (0.87–1.23; 0.68)	0.97 (0.94–1.00; 0.52)	0.93 (0.89–0.97; 0.35)	0.95 (0.79–1.14; 0.65)
DCIS margin close or positive	1.01 (0.91–1.12; 0.79)	1.00 (0.93–1.08; 0.95)	1.00 (0.90–1.12; 0.93)	1.01 (0.87–1.18; 0.89)
Invasive *or* DCIS margin close or positive	1.04 (0.95–1.14; 0.34)	0.98 (0.93–1.04; 0.61)	1.00 (0.92–1.09; 0.99)	1.00 (0.88–1.14; 0.98)

Data are presented as relative risk (95% confidence interval; p-value [Chi-squared test for two independent proportions])

Final histologic margins in patients undergoing skin- or nipple-sparing mastectomy and immediate implant-based reconstruction for breast cancer at North Canberra Hospital, Australian Capital Territory, Australia; March 2012 to September 2022. For invasive carcinoma and DCIS, positive margins were 0 mm. For invasive carcinoma, close margins were <1 mm. For DCIS, close margins were <2 mm; DCIS, ductal carcinoma in situ

To detect potential confounders, binomial logistic regression analysis was performed. Associations between margin status and recurrence when receiving adjuvant radiotherapy, chemotherapy, or endocrine therapy were tested. The results are summarized in Table 4. When any anterior margin was positive or close, radiotherapy appeared to significantly reduce the rates of ipsilateral breast cancer recurrence. P-values for odds ratios (ORs) were <0.05 across all these domains (Table 4). When the anterior margin was positive and/or close, the addition of radiotherapy appeared to reduce the risk of recurrence by a factor of 13 times (OR 0.08; 95% CI 0.01–0.71, *p* = 0.02).

**Table 4:** Margin and recurrence adjusting for confounding factors

Margin and recurrence	Ipsilateral recurrence	Locoregional recurrence	Distant metastasis
Invasive margin positive			
Chemotherapy	0.58 (0.15–2.03), 0.44	0.90 (0.28–2.84), 0.85	2.06 (0.42–10.14), 0.37
Radiotherapy	0.09 (0.01–0.85), 0.04	0.36 (0.10–1.23), 0.10	2.47 (0.60–10.05), 0.21
Endocrine therapy	0.26 (0.05–1.23), 0.09	0.60 (0.19–1.93), 0.39	1.26 (0.31–5.23), 0.75
Invasive margin positive or close			
Chemotherapy	0.55 (0.13–2.22), 0.40	0.83 (0.25–2.67), 0.75	1.81 (0.36–9.14), 0.47
Radiotherapy	0.09 (0.01–0.76), 0.03	0.31 (0.08–1.09), 0.07	2.14 (0.51–8.92), 0.30
Endocrine therapy	0.25 (0.06–1.17), 0.08	0.54 (0.17–1.73), 0.30	1.10 (0.26–4.56), 0.90
DCIS margin positive			
Chemotherapy	0.59 (0.15–2.29), 0.44	0.86 (0.27–2.70), 0.79	1.98 (0.40–9.71), 0.40
Radiotherapy	0.10 (0.01–0.88), 0.04	0.34 (0.10–1.14), 0.08	2.25 (0.55–9.13), 0.26
Endocrine therapy	0.29 (0.07–1.27), 0.10	0.58 (0.19–1.83), 0.36	1.26 (0.31–5.09), 0.75
DCIS margin positive or close			
Chemotherapy	0.30 (0.06–1.29), 0.10	0.86 (0.27–2.71), 0.80	2.02 (0.41–9.86), 0.39
Radiotherapy	0.10 (0.01–0.86), 0.04	0.34 (0.10–1.15), 0.08	2.37 (0.59–9.61), 0.23
Endocrine therapy	0.30 (0.07–1.29), 0.11	0.58 (0.19–1.84), 0.36	1.23 (0.30–4.98), 0.77
Invasive or DCIS margin close or positive			
Chemotherapy	0.54 (0.14–2.13), 0.38	0.86 (0.27–2.71), 0.80	2.04 (0.41–10.02), 0.38
Radiotherapy	0.08 (0.01–0.71), 0.02	0.31 (0.09–1.09), 0.07	2.45 (0.60–10.08), 0.21
Endocrine therapy	0.26 (0.06–1.18), 0.08	0.56 (0.18–1.76), 0.32	1.23 (0.30–4.97), 0.77

Data are presented as odds ratio (95% confidence interval), *p*-value (binomial logistic regression)

Final histologic margins in patients undergoing skin- or nipple-sparing mastectomy and immediate implant-based reconstruction for breast cancer at North Canberra Hospital, Australian Capital Territory, Australia; March 2012 to September 2022. For invasive carcinoma and DCIS, positive margins were 0 mm. For invasive carcinoma, close margins were <1 mm. For DCIS, close margins were <2 mm; DCIS, ductal carcinoma in situ

### Margin Status and Radiotherapy

In this study, patients with close or positive margins were more likely to be managed with adjuvant radiotherapy (ꭕ^2^ RR 1.7; 95% CI 1.09–2.58; *p* 0.009).

### Margin Status and Neoadjuvant Chemotherapy

Administration of neoadjuvant chemotherapy did not appear to reduce the risk of close positive margins (ꭕ^2^ RR 0.79; 95% CI 0.66–0.96; *p* 0.47).

### Implant-Related Outcomes

The implant loss rate within 3 months was 8% (13/160). During the study period, there was exchange or loss of the initial implant in a total 71 patients (44%).

Five patients who did not receive radiotherapy, and eight who did, lost implants in the first 3 months following surgery. The most common indication for further implant-related surgery was capsular contracture. Of the 71 patients who had their implants revised or removed in the longer term, 47 (66%) had received radiotherapy. Using Chi-squared analysis, the use of radiotherapy was associated with increased overall rates of exchange or loss of the initial implant over the study period (ꭕ^2^ RR 1.65; 95% CI 1.23–2.22; *p* < 0.001).

## Discussion

In this study, close or involved margins on final pathology after SSM/NSM and implant-based breast reconstruction were not uncommon. In most cases, these margins were not re-excised. There was no statistically significant association between margin status and recurrence rates. Patients with involved or close margins were more likely to receive PMRT, and the addition of radiotherapy reduced rates of ipsilateral breast cancer recurrence. However, radiotherapy was also associated with increased overall rates of unplanned exchange or loss of the initial breast implant.

After SSM/NSM, the identification of positive or close anterior margins on pathological examination represents a difficult clinical scenario. Despite advances in imaging and careful preoperative patient selection, positive anterior margins can arise because of several factors. These include the final histologic disease extent, which can differ from the pre-operative imaging assessment. Additionally, the removal of all breast tissue can be difficult because of a discontinuous or imperceptible plane between breast and subcutaneous tissue ^7,8,18–21^ and the substantial variability in individual skin flap thickness.^21^ The incision can also limit surgical exposure,^7,8,20^ and intra-operative shaves are open to sampling error.^18,22^ Attempts to preserve flap blood supply, enable longer flaps, or improve cosmesis (such as to reduce wrinkling or “steps”), all bias toward thicker flaps, leading to greater likelihood of residual breast tissue.^19^ Published data indicate that up to 7.3% of breast tissue can remain after SSM/NSM,^23^ which may represent a greater residual volume than that following simple mastectomy.^24^ A recent study utilizing MRI calculated the median residual breast and skin volumes at 368 mL.^23^ Higher residual breast tissue volume may predispose to positive or close margins.

Positive margins may be more common than previously thought. Studies have shown that positive or close margins may be twice as likely in patients undergoing SSM/NSM than in those undergoing simple mastectomy.^4,5,25^ Literature review shows positive anterior margins occur in 4.5–38% of SSM/NSM procedures.^12,18,21,26,27^ This held true for this series, with 19% of patients having a positive anterior margin and a further 11% (*n*=18) having close anterior margins.

Recurrence, even when locoregional, generally leads to reductions in overall survival^28^ and increased healthcare-associated costs. Quality indicators defining acceptable locoregional recurrence rates are not well established. Population-based audit quality assurance measures in the Netherlands for any breast cancer surgery showed that the cumulative risk of locoregional recurrence was 3.3% over 5 years.^29^ The locoregional recurrence rate in this series was higher, at 9% (*n*=14) over a mean 5.3 years and may reflect the more locally advanced cancers in our SSM/NSM population.

There is a long-held assumption of oncologic equivalence between simple mastectomy and SSM/NSM, although this is based on low‐certainty evidence from observational studies.^1^ In this study, there was no clear evidence that close or involved anterior margins resulted in increased rates of breast cancer recurrence. This study had several limitations. Interpretation of findings was limited by the small sample size. This lack of significant effect appears in part due to effective adjuvant therapy, particularly radiotherapy. Our mean follow-up period was 5.3 years, and longer follow-up may influence overall recurrence rates. There is variability in the literature as to the likely median time to recurrence after SSM/NSM, with some stating it is more likely as early as 24 months^12,23^ and some as late as 8 years.^25^ A total of 8% of patients were lost to follow-up. The retrospective nature of study could not account for patient and provider decision-making about adjuvant treatments.^4,5^

Data to guide clinical management of this situation are lacking and vary in international clinical practice.^30^ Multidisciplinary team involvement is recommended, and therapeutic options include re-excision, PMRT, or close surveillance with imaging.

### Re-Excision

The current US NCCN guidelines state that re-excision is preferred. However, accurate localization of the site of margin involvement can be difficult, ^4,5,7,8,12,20,31^ and a return to theatre for margin revision may delay adjuvant therapies.^32–34^ Further surgery may risk compromising lipo-dermal flap viability, increase the risk of implant infection, have negative effects on aesthetic outcomes,^4,5^ lead to reconstruction failure,^12^ and pose economic^35^ and psychologic impacts. These challenges highlight the importance of careful preoperative planning and attention to patient consent and counselling before SSM/NSM and breast reconstruction.^19^

### Radiotherapy

We have shown a statistically significant reduction in rates of ipsilateral recurrence, in the setting of positive anterior margins, when adjuvant radiotherapy is administered. The treatment effect was sizeable (OR 0.08). The omission of radiotherapy when an anterior margin was involved with either invasive breast cancer and/or DCIS increased the odds of recurrence by a factor of 13. This significant reduction in risk of local recurrence is consistent with the randomized evidence for PMRT in the broader setting.^36,37^ This supports current guideline recommendations. The current NCCN guidelines recommend consideration of radiotherapy when involved margins cannot be surgically excised.^14^ Similarly, the guidelines published by the American Society for Radiation Oncology, Society for Surgical Oncology, American Society of Clinical Oncology, UK National Institute for Health and Care Excellence, and European Society for Medical Oncology include involved margins after mastectomy as an indication for PMRT,^7,8,10,38^ although without specific reference to SSM/NSM techniques. In the setting of close but not positive margins, the indications for PMRT remain unclear because adequate margin width recommendations after mastectomy remain poorly defined.

Our results are supported in the literature. A 2011 study reported a fivefold higher rate of local recurrence following all types of mastectomy, when positive margins were present, and PMRT was omitted.^11^ A 2024 study^12^ also found that PMRT reduced the incidence of LR in patients with breast cancer with margin involvement after mastectomy via simple or SSM/NSM techniques. The 7-year cumulative incidences of LR were 1.9% with radiotherapy and 12.6% without radiotherapy (hazard ratio 0.17; 95% CI 0.04–0.80; *P* 0.025).^12^ Combined with our results, these data provide consistent and increasingly compelling evidence supporting the use of radiotherapy in this context.

PMRT should not be used routinely to salvage patients who have had inadequate surgery because sequalae of treatment can lead to significant complications, reduced aesthetic outcomes, and implant loss in the long term.^23,24,39,40^ In this study, use of radiotherapy strongly correlated with either a need for implant exchange or loss of the initial implant. This was often a late complication due to side effects such as capsular contracture. When PMRT is indicated solely to manage a positive anterior margin, it may mitigate the quality-of-life benefits associated with attempts at reconstruction in the first place.^25^ In this setting, further surgery should be considered if PMRT might be avoided.

### Surveillance

There is no accepted standard for surveillance for the reconstructed breast after SSM/NSM with positive or close margins. In our institution, annual breast ultrasound or breast MRI was performed.

### Prevention

Prevention of postoperative anterior margin involvement involves careful patient selection, utilization of imaging modalities including breast MRI to carefully plan surgery, and the downstaging of appropriate patients with neoadjuvant chemotherapy. Other surgical strategies include the increased use of level two oncoplastic techniques, which may avoid the need for mastectomy. Certain tumor characteristics, such as multifocality and lymphovascular invasion may predispose to close margins.^22^ Informed consent cannot be overemphasized.^15,41^ In some centers, PMRT has been cited as a contraindication to implant-based breast reconstruction.^36^ We routinely offer implant-based reconstruction to appropriately consented patients requiring mastectomy, even when PMRT is likely to be required, with the option of considering tissue reconstruction once oncological treatments are complete.

In conclusion, further evidence from large multicenter studies is required to guide safe oncologic management of close of positive anterior margins after SSM/NSM. Quality assurance programs should recommend clear margins, and cases should be discussed with a multidisciplinary team. Surgery for re-excision should be considered if PMRT could otherwise be avoided. However, PMRT may be a safe oncologic alternative when re-excision is not possible. Patients and clinicians need to accept the attendant aesthetic risks and increased need for further revision surgeries to support improved breast reconstruction outcomes in these patients.
